# Clinical characteristics of pediatric acute epididymitis: a single institute review of 142 cases

**DOI:** 10.1007/s00383-025-06168-7

**Published:** 2025-08-30

**Authors:** Ryoya Furugane, Tetsuya Mitsunaga, Shugo Komatsu, Ayako Takenouchi, Satoru Oita, Yunosuke Kawaguchi, Wataru Kudo, Katsuhiro Nishimura, Tomoro Hishiki

**Affiliations:** Department of Pediatric Surgery, Graduate School of Medicine, Chiba University, 1-8-1 Inohana, Chiba, 260-8677 Japan

**Keywords:** Pediatric, Acute epididymitis, Risk factors, Testicular necrosis, Testicular atrophy

## Abstract

**Purpose:**

Acute epididymitis (AE) is a common cause of scrotal pain and swelling in children; however, its etiology and risk factors for poor outcomes remain unclear. This study aimed to identify the clinical characteristics and potential risk factors associated with poor AE outcomes.

**Methods:**

We retrospectively reviewed pediatric patients with AE treated at our hospital. Clinical data, laboratory results, ultrasonographic findings, and treatment strategies were analyzed. We conducted comparative analyses to identify significant risk factors linked to poor outcomes.

**Results:**

A total of 142 patients with a median age of 9 years were reviewed. Of these, 137 cases had favorable testicular outcomes, while five cases had poor outcomes, including three cases of testicular or epidydimal atrophy, one case of testicular necrosis, and one case of testicular necrosis and abscess. Fever (p = 0.001), C-reactive protein (CRP) levels (p = 0.002), and decreased testicular blood flow on ultrasonography (p < 0.001) were significantly associated with poor testicular outcomes.

**Conclusion:**

Fever, CRP levels, and decreased testicular blood flow are risk factors for unfavorable testicular outcomes in pediatric patients with AE. Patients with these risk factors require careful monitoring and may benefit from aggressive management strategies.

## Introduction

Acute epididymitis (AE) is an infection or inflammation of the epididymis that causes scrotal pain and swelling [1]. AE is the primary causes of acute scrotum, along with testicular torsion, testicular appendix torsion, and orchitis, and is relatively common [2, 3]. Testicular torsion requires emergency surgery and can be challenging to differentiate from AE.

In adults, AE is frequently associated with sexually transmitted infections in those aged 20–39 years, whereas in older patients, ascending urinary tract infections, particularly those involving *Escherichia coli* (*E. coli*) and other coliform bacteria, are the predominant cause [4]. Therefore, antibiotic treatment is generally required to target specific pathogens. In contrast, pediatric AE often presents with low rates of positive urinalysis and urine cultures [5, 6], and the underlying etiology remains unclear. Some reports suggest that antibiotic treatment may not be necessary in cases without pyuria or bacteriuria [5, 6]; however, there is no consensus on the necessity and optimal duration of observation.

Pediatric AE typically resolves without complications. However, some cases progress to poor testicular outcomes, including testicular atrophy, abscess, or necrosis, necessitating orchiectomy [7, 8]. Identifying factors associated with poor outcomes is crucial for improving clinical management. Recent studies have suggested that elevated levels of inflammatory markers such as C-reactive protein (CRP) and abnormal findings on ultrasonography may be linked to poor testicular outcomes [9, 10], although evidence remains limited. In addition, despite the low incidence of positive urine cultures, antibiotic use remains prevalent, raising questions regarding the necessity and appropriateness of empirical antibiotic therapy. In this study, we retrospectively reviewed pediatric AE cases to elucidate their clinical characteristics and investigate potential risk factors for poor outcomes. Furthermore, we investigated the relationship between the positivity rates of urinary white blood cell (U-WBC) and urine cultures, antibiotic usage rate, and their association with poor outcomes.

## Materials and methods

### Patients

We retrospectively reviewed 142 cases of AE in patients aged 0–15 years who were treated at our department between 2002 and 2024. All patients were diagnosed based on clinical symptoms such as scrotal pain, swelling, and redness, along with Doppler ultrasonography findings of epididymal enlargement and increased blood flow. Urinalysis, urine culture, blood tests, voiding cystourethrography (VCUG), and intravenous pyelography (IP) were performed as required. For patients with recurrent episodes, only the data from the most recent episodes were included. Patients with scrotal pain due to other conditions, those with long-term indwelling urological devices, and immunocompromised patients were excluded.

### Data collection

Clinical information was collected from electronic medical records, including age at diagnosis, affected side, urological comorbidities, time from onset to presentation, initial physical findings (scrotal pain, swelling, redness, fever,which was defined as a body temperature of ≥ 37.5 ℃), initial ultrasonographic findings, and initial laboratory data such as white blood cell count (/μL), serum CRP (mg/dL), U-WBC (cells/high-power field:HPF), urine culture results, treatment details, period from the initial diagnosis of AE to the final follow-up, and for cases undergoing VCUG, IP, or surgery, imaging findings, surgical details, and pathology findings.

**Fig. 1:**
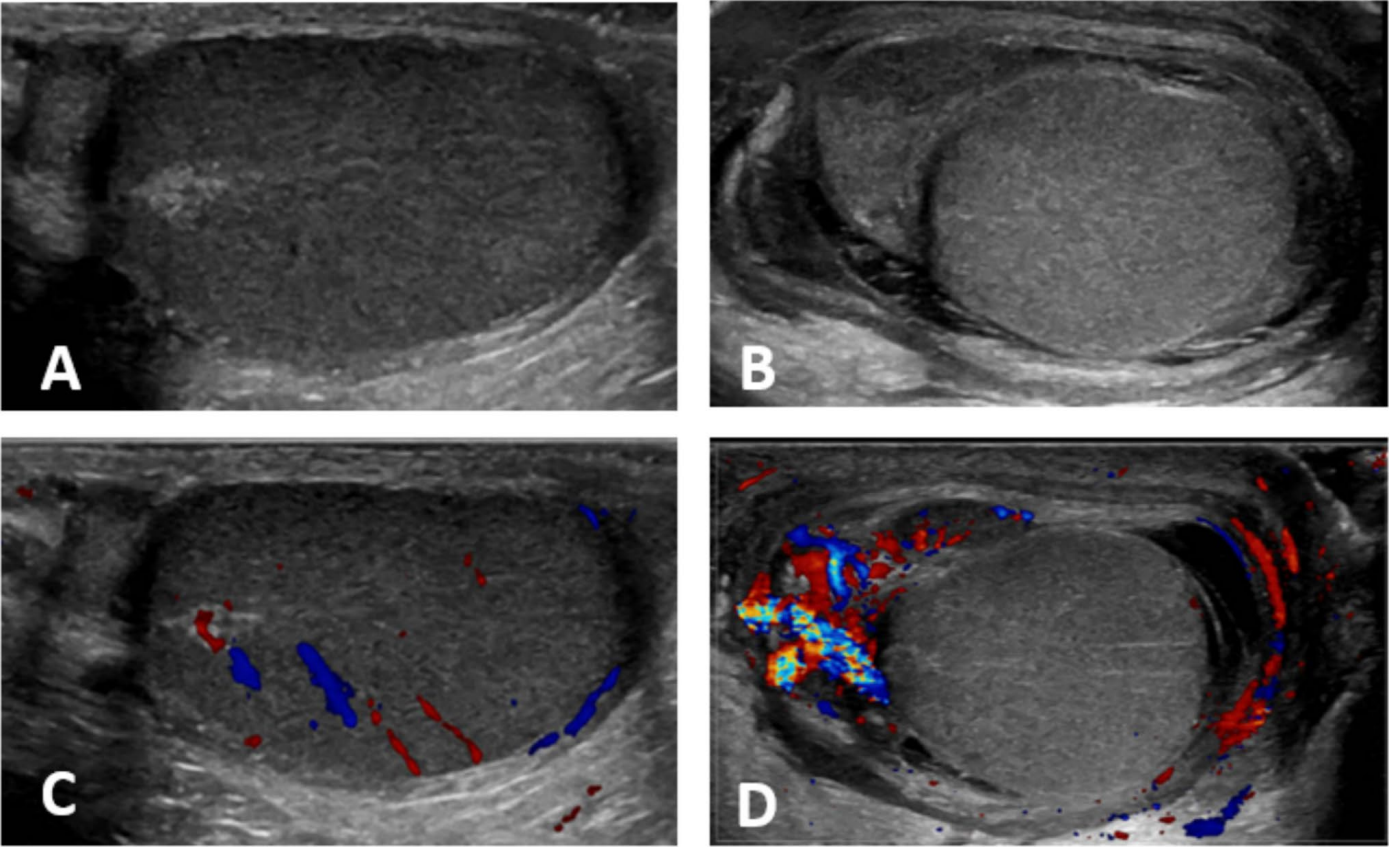
Representative ultrasonography images of epididymitis. Compared to the left side in **A** and **C**, the right epididymis in **B** and **D** is enlarged and shows increased blood flow. Additionally, decreased blood flow is observed in the right testis

Ultrasonography was performed by pediatric surgeons with sufficient experience in pediatric scrotal ultrasonography. Epididymal enlargement and increase or decrease testicular/epididymal blood flow were evaluated in comparison with the contralateral side (Fig. 1). These findings were considered positive only when explicitly documented in the ultrasonography report.

Urine samples for urinalysis and urine culture were collected using either the clean-catch method or catheterization, depending on the patient’s age. Cases with a U-WBC count of > 5 cells/HPF were considered positive. A positive urine culture was defined as the detection of a single bacterial species at ≥ 1.0 × 10^3^ colony-forming units (CFU) per mL in catheterized urine, or ≥ 1.0 × 10^4^ CFU/mL in clean-catch urine [11, 12].

### Analysis

To identify potential risk factors associated with poor outcomes, patients were classified into favorable outcome (group A) and poor outcome (group B) groups, and a comparative analysis was performed. Poor outcomes were defined as cases in which orchiectomy was performed or in which testicular or epididymal atrophy occurred during post-treatment follow-up, as these conditions are expected to significantly affect future testicular function and fertility [13–16]. Continuous variables were summarized using the median and interquartile range (IQR) unless otherwise noted. Comparisons between the two groups were performed using Fisher’s exact test for categorical variables or the Mann–Whitney U test for continuous variables. Statistical significance was defined as a two-sided p < 0.05. All statistical analyses were performed using the Python software (version 3.11). Analyses were conducted after excluding patients with missing data.

### Ethics

This study was conducted in accordance with the ethical guidelines for medical research in Japan and the principles of the Declaration of Helsinki. This study was approved by the Ethics Committee of Chiba University Hospital in September 2021 (M10108). The requirement for informed consent was waived because the study design was retrospective, and participant privacy was ensured.

## Results

### Patient background

**Fig. 2:**
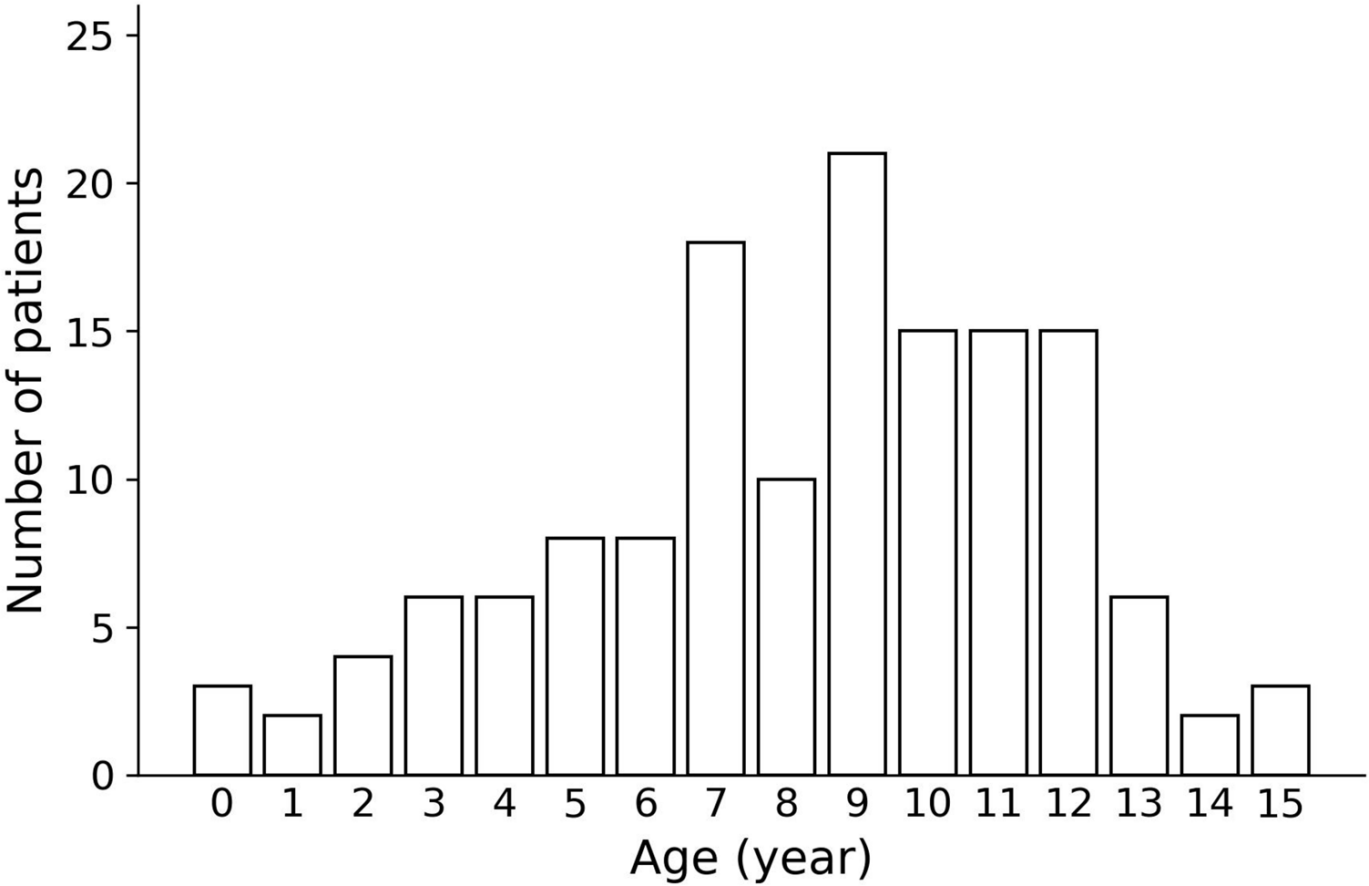
Age distribution of patients with acute epididymitis

The distribution of patient ages is shown in Fig. 2. The median age at presentation was 9 years (interquartile range [IQR]: 6–11 years). Prepubertal children (8–12 years old) accounted for approximately half of all cases (76 cases; 53.5%). The affected side was almost evenly distributed between the right (73 cases) and the left (68 cases), with one bilateral case (Table 1).

**Table 1:** Clinical and laboratory characteristics and treatment of patients with acute epididymitis

	All patients (n = 142)	Favorable outcome (Group A, n = 137)	Poor outcome (Group B, n = 5)	*p* value
Age, years	9 (6–11)	9 (6–11)	9 (3–9)	0.55*
Laterality				0.20
Right	73 (51.4)	72 (52.6)	1 (20.0)	
Left	68 (47.9)	64 (45.1)	4 (80.0)	
Bilateral	1 (0.7)	1 (0.7)	0 (0)	
Urological comorbidity	9 (6.3)	9 (6.6)	0 (0)	1.0
Time from onset to presentation (hours)	20 (7–44)	19.5 (7–38)	48 (48–72)	0.08*
Symptoms/physical findings				
Scrotal pain	139 (97.9)	134 (97.8)	5 (100.0)	1.0
Scrotal swelling	79 (55.6)	76 (55.5)	3 (60.0)	1.0
Scrotal redness	63 (44.4)	60 (43.8)	3 (60.0)	0.65
Fever	9 (6.3)	6 (4.4)	3 (60.0)	0.001
Laboratory findings				
U-WBC > 5 cells/HPF, n = 95	5 (5.2)	5 (5.5)	0 (0)	1.0
Positive result of urine culture, n = 48	2 (4.2)	2 (4.5)	0 (0)	1.0
WBC (10^3^ count /μL), n = 70	8.50 (6.93–10.50)	8.50 (7.03–10.50)	12.90 (10.20–17.90)	0.15*
CRP (mg/dL), n = 71	0.10 (0.00–0.30)	0.10 (0.00–0.30)	0.72 (0.53–1.81)	0.002*
Ultrasonography findings				
Epididymis enlargement	115 (81.0)	111 (81.0)	4 (80.0)	1.0
Increased blood flow in the epididymis	123 (86.6)	119 (86.9)	4 (80.0)	0.52
Increased blood flow in the testis	34 (23.9)	33 (24.1)	1 (20.0)	1.0
Decreased blood flow in the testis	3 (2.1)	0 (0)	3 (60.0)	< 0.001
Effusion around testis	19 (13.3)	18 (13.1)	1 (20.0)	0.52
Abnormal finding of VCUG and IP, n = 5	0 (0)	0 (0)	0 (0)	1.0
Antibiotic therapy				
Oral	40 (28.2)	38 (27.7)	2 (40.0)	0.62
Intravenous	54 (38.0)	52 (38.0)	2 (40.0)	1.0
Surgery	3 (2.1)	0 (0)	3 (60.0)	< 0.001

Values are presented as median (interquartile range) or number (%). *p* values are calculated using Fisher’s exact test (without asterisks) or the Mann–Whitney U test (with asterisks)

*U-WBC* urinary white blood cell, *HPF* high-power field, *WBC* white blood cell, *CRP* C-reactive protein, *VCUG* voiding cystourethrography, *IP* intravenous pyelography

### Clinical characteristics of patients with favorable or poor testicular outcomes

Of the 142 cases, 137 were categorized into Group A, whereas five (orchiectomy, n = 2; testicular atrophy, n = 2, epididymal atrophy, n = 1) were categorized into Group B. Clinical findings, laboratory results, and treatment details of the patients in the two groups are summarized in Table 1. The most common symptoms and physical findings were scrotal pain, followed by swelling and redness of the scrotum. Fever was observed significantly more frequently in Group B compared to Group A. Urinalysis and urine culture were performed in a subset of patients. The positivity rates for urinalysis (U-WBC) were 5.5% in Group A and 0% in Group B. For urine culture, the positivity rates were 4.5% in Group A and 0% in Group B. Notably, two infants in Group A had positive urine cultures: a 3-month-old infant (catheterized urine, *E. coli* 1.0 × 10^4^ CFU /mL) and, a 4-month-old infant (clean-catch urine, *E. coli* 1.0 × 10^4^ CFU/mL). As for blood tests, the WBC count tended to be higher in Group B, but no significant difference was observed (Table 1). Group B had significantly higher CRP levels than Group A (median 0.72 vs. 0.10). Ultrasonography revealed an increased epididymal blood flow in 86.9% of Group A and 80.0% of Group B, and epididymal enlargement in 81.0% and 80.0%, respectively. Decreased testicular blood flow was noted in three cases, all in Group B. VCUG or IP was performed in five cases due to recurrent epididymitis (n = 2), hydronephrosis (n = 1), or urinary tract infection (n = 2). No underlying urinary tract abnormalities, such as urethral valves, urethral strictures, or ectopic ureteral openings, were identified in the patients who underwent VCUG and IP. Regarding the use of antibiotics, there was no significant difference in the usage rates of both intravenous and oral antibiotics between the two groups. The median observation period was 14 days (7–48.5) for the entire cohort. Group B had a longer observation period (median: 407 days, IQR: 365–998) compared to Group A (median: 12 days, IQR: 7–35).

**Fig. 3:**
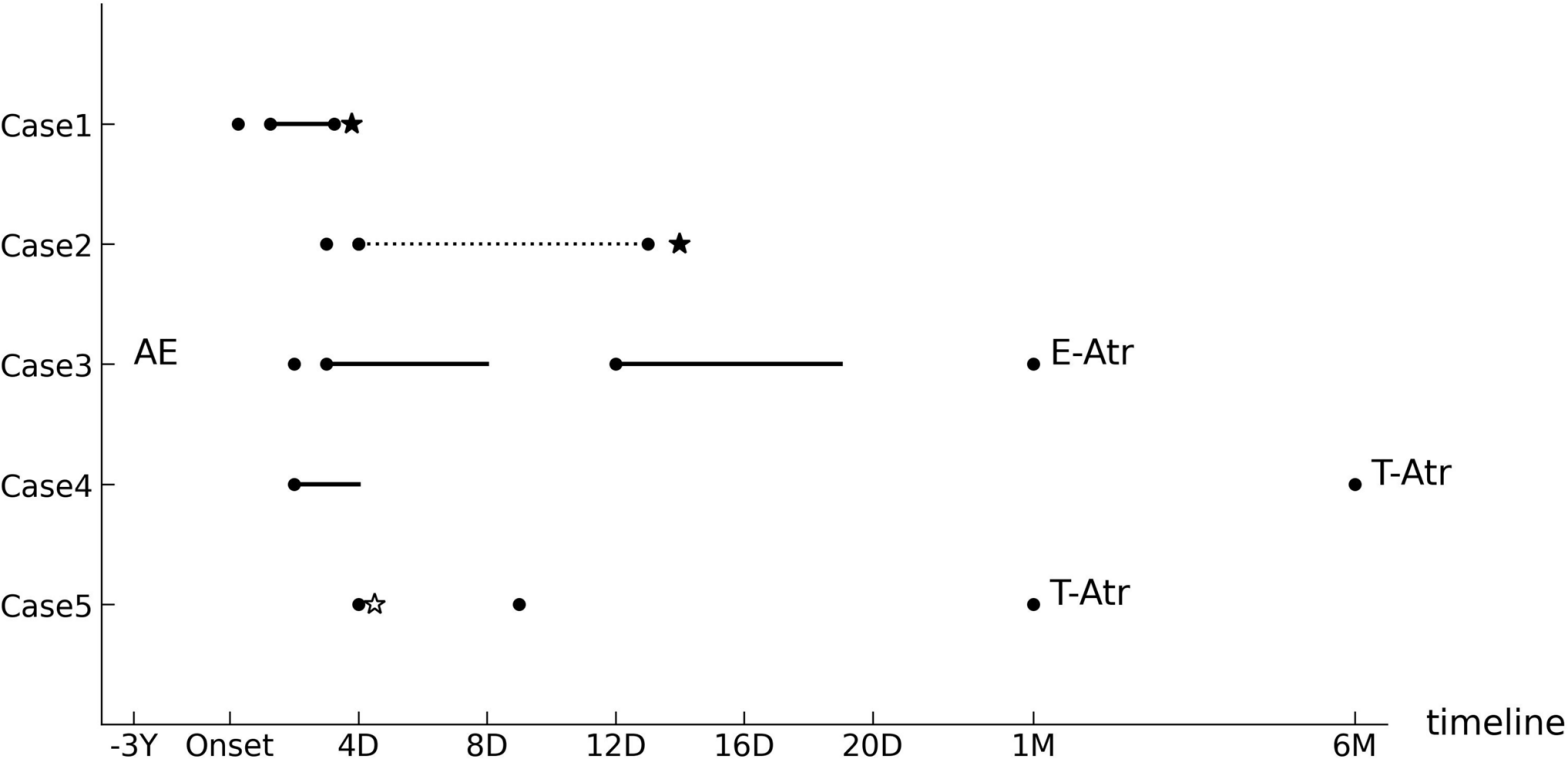
Treatment timeline of poor outcome cases until the diagnosis of poor outcome. The vertical axis represents the cases, and the horizontal axis represents the timeline. *Y* year, *D* day, *M* month, *Onset* acute epididymitis onset, ● hospital visit, ― antibiotic treatment, --- prednisolone treatment, ☆ orchidopexy, ★ orchiectomy, *AE* episode of acute epididymitis, *T-Atr* testis atrophy, *E-Atr* Epididymis atrophy

The initial clinical findings and laboratory results for patients in Group B are summarized in Table 2, and their clinical course is summarized in Fig. 3. Two patients developed testicular abscesses or necrosis in the acute phase, requiring drainage and orchiectomy, whereas three later developed testicular or epididymal atrophy. Details of each case are as follows.

**Table 2:** Clinical and laboratory characteristics of patients with severe acute epididymitis

Case no.	1	2	3	4	5
Age, years	3	9	9	13	1
Laterality	Left	Right	Left	Left	Left
Urological comorbidity	−	−	−	−	−
Time from onset to presentation (hours)	5	72	48	48	96
Symptoms / Physical findings					
Scrotal pain	+	+	+	+	+
Scrotal swelling	+	+	−	−	+
Scrotal redness	+	+	−	−	+
Fever	+	+	−	+	−
Laboratory findings					
U-WBC cells /HPF	0	0	0	N/A	0
Result of urine culture	Negative	Negative	Negative	N/A	Negative
WBC( 10^3^ count /μL)	11.6	29.0	N/A	6.0	14.2
CRP (mg/dL)	0.88	4.59	0.56	0.42	1.41
Ultrasonography findings					
Epididymis enlargement	+	+	+	+	−
Blood flow in the epididymis	Increased	Increased	Increased	Increased	No change
Blood flow in the testis	Decreased	Decreased	No change	Increased	Decreased
Effusion around testis	−	+	−	−	−
VCUG and IP	N/A	N/A	N/A	N/A	N/A
Antibiotic therapy	Oral	N/A	Oral	Intravenous	Intravenous
Surgery	Orchiectomy	Orchiectomy	N/A	N/A	Orchidopexy
Observation period (days)	365	407	998	183	2200

*U-WBC* urinary white blood cell, *HPF* high-power field, *WBC* white blood cell, *CRP* C-reactive protein, *VCUG* voiding cystourethrography, *IP* intravenous pyelography, *N/A* not applicable

### Cases undergoing orchiectomy

#### Case 1

A 3-year-old boy presented to our department 5 h after symptom onset and was diagnosed with left epididymitis. Upon arrival, ultrasonography revealed enlargement and increased blood flow of the epididymis, along with decreased blood flow to the affected testis. Antibiotic therapy was initiated. On the third day of treatment, the pain worsened, and follow-up ultrasonography revealed no detectable blood flow in the affected testis. Emergency surgery was performed after the diagnosis of testicular necrosis. The testis appeared dark red, and intraoperative indocyanine green examination confirmed the absence of blood flow, leading to orchiectomy. There was no evidence of testicular torsion. Histopathological findings revealed inflammatory cell infiltration in the epididymis and significant hemorrhage in the testicular interstitium.

#### Case 2

A 9 year-old boy presented 72 h after symptom onset. In addition to right scrotal pain and swelling, he presented with abdominal pain and wheals. Blood tests showed an elevated inflammatory response with WBC 29.0 × 10^3^ count/µL and CRP 4.59 mg/dL. Ultrasonography revealed enlargement and increased blood flow of the right epididymis, as well as effusion around the testis. These findings, along with his clinical symptoms, raised the suspicion of IgA vasculitis. Prednisolone was administered intravenously (1 mg/kg per day) for 9 days, and then switched to oral prednisolone. However, his scrotal pain recurred the following day. Ultrasonography revealed a discontinuity of the tunica albuginea and hypoechoic fluid collection around the testis, suggesting a testicular abscess that prompted emergency surgery. Pus drainage was observed in the right testis, with minimal remaining testicular tissue, leading to orchiectomy. *Pseudomonas aeruginosa* was isolated from the abscess cultures. There was no evidence of testicular torsion. Histopathology revealed intense inflammatory cell infiltration in the epididymis.

### Cases with testicular or epididymal atrophy

#### Case 3

A 9-year-old boy was treated for epididymitis 3 years prior to visiting our department. He presented with mid pain 48 h after symptom onset, for which analgesics were prescribed. However, the following day, his scrotal pain worsened, prompting a re-evaluation. Ultrasonography revealed worsening inflammation, and a 5-day course of antibiotics was initiated. Three days after completing treatment, he experienced recurrent scrotal pain. Ultrasonography revealed a mosaic-like change within the left epididymis, leading to an additional 7-day course of antibiotics treatment. Ultrasonography confirmed epididymal atrophy 1 month after onset.

#### Case 4

A 13-year-old boy received a mumps vaccine 3 weeks before developing left scrotal pain. Although no erythema or swelling was present, he had a high fever of 39.2 °C. Ultrasonography revealed left epididymal enlargement with increased blood flow. Antibiotic treatment was administered for 2 days, which led to symptom improvement. However, follow-up ultrasonography at 6 months confirmed the presence of testicular atrophy.

#### Case 5

A 1-year-old boy presented 96 h after symptom onset. Ultrasonography revealed reduced blood flow in the testis and epididymis, raising the suspicion of testicular torsion and prompting emergency surgery. Intraoperatively, no twisting of the spermatic cord was observed. However, the left epididymis was enlarged and adhered to the surrounding serosa, leading to a diagnosis of epididymitis. A left orchiopexy was performed. Follow-up ultrasonography performed 1 month postoperatively confirmed testicular atrophy.

## Discussion

This retrospective analysis included 142 patients with AE who were treated in our department. Similar to previous reports [7, 17], most patients were prepubertal. While some studies have reported a bimodal distribution with a peak in infancy [7, 18], our cohort exhibited a gradual peak between the ages of 7 and 12 years, with a low proportion of infants, accounting for only 15 cases (10.6%). This trend differs from reports from other regions and institutions, possibly due to the characteristics of our patient population and their relationships with the surrounding hospitals.

The positive rate of the U-WBC count on urinalysis was low (5.2%), and the number of urine culture-positive cases was also limited (4.2%). A recent meta-analysis study identified 1496 pediatric patients with AE and reported that only 10.8% of patients with comprehensive data had a positive urine culture [19]. Our findings are consistent with those of this meta-analysis and confirm that urinalysis and urine culture results are often negative. Additionally, a study reported that 26.4% of pediatric AE cases had underlying renal and urinary tract anomalies, with a positive urine culture being the only predictor [20]. In our cohort, VCUG was performed for recurrent cases, cases with associated hydronephrosis, and urine culture-positive cases; however, no renal or urinary tract anomalies were identified.

The positive rate of urinalysis and urine culture in pediatric AE is also relevant to discussions regarding antibiotic use. Previous studies have suggested that owing to the low positivity rates of urinalysis and urine culture, antibiotic use may not be essential for prepubertal AE [5, 6]. However, in practice, antibiotics are commonly used, with the aforementioned meta-analysis reporting an antibiotic usage rate of 81.7% [19]. In our cohort, the antibiotic usage rate was 66.2%, which was slightly lower than that in the meta-analysis, but still high. This result reflects treatment strategies that consider the risk of poor outcomes and complications. Moving forward, more precise criteria are required to optimize antibiotic use.

AE is generally considered to have minimal long-term impact on testicular function [6, 21]; however, in the acute phase, testicular necrosis and abscess formation can occur [22, 23], and some cases of delayed testicular atrophy after treatment have been reported [7, 24]. In our study, the poor testicular outcomes tended to have prolonged intervals between onset and hospital visits, suggesting that delayed diagnosis and treatment contributed to disease progression and irreversible damage to the testis. Poor outcomes were characterized by fever, CRP levels in blood tests, and decreased testicular blood flow on ultrasonography, indicating that these factors may serve as risk predictors of poor outcomes. While AE typically does not present with systemic signs such as fever or elevated CRP levels, these findings may emerge when local inflammation is particularly severe. In our study, the median CRP level in the poor outcome group was relatively low at 0.72 mg/dL; however, there was a statistically significant difference between the groups, indicating that even slight elevations in CRP may warrant clinical attention. Based on these results, in cases of AE, patients with fever or relatively severe epididymitis findings on ultrasonography should undergo blood tests in addition to urinalysis. If CRP levels are slightly elevated or pyuria is present, antibiotic therapy should be considered.

Testicular and epididymal atrophy is thought to be caused by venous congestion due to inflammatory edema, which leads to thrombosis and ischemia [25, 26]. In case 3 showed mosaic-like changes in the epididymis on ultrasonography, which were indicative of a partial infarction. This may have been caused by venous congestion due to inflammatory edema, leading to thrombus formation. However, pathological confirmation was not possible because the patient did not undergo surgery. Additionally, the mechanisms testicular atrophy following epididymitis include the spread of inflammation to the testis; in adult cases, it has been reported that testicular compartment syndrome occurs due to swelling of the epididymis and testis, which is known to cause testicular ischemia [27]. In our cohort, Case 5 in the poor outcome group exhibited intraoperative findings of testicular swelling and adhesions to the surrounding serosa, suggesting inflammatory spread to the testis. Furthermore, case 1 and case 2 showed reduced testicular blood flow on ultrasonography, raising the possibility of compartment syndrome. To prevent compartment syndrome, close monitoring via ultrasonography is necessary, and if signs of compartment syndrome are detected, surgical intervention such as tunica albuginea decompression incision should be performed at an appropriate time [27, 28]. Although still at the research stage using rat models, antioxidant therapy has been reported to potentially be effective in preventing testicular damage caused by inflammation, and further research is expected [29, 30]. These strategies may be beneficial in improving outcomes, particularly in severe cases.

In Case 2, epididymitis progressed to testicular abscess, necessitating orchiectomy. Initially, IgA vasculitis was suspected, and intravenous steroid administration was performed without antimicrobial therapy. Although symptoms were temporarily improved by the analgesic effect of steroids, inflammation of the affected testis progressed, ultimately leading to abscess formation. It is unclear whether steroid administration caused abscess formation, but it may have masked symptoms and delayed intervention. Based on the high inflammatory markers at the initial visit, the addition of antibiotics should have been considered. However, *Pseudomonas aeruginosa* was detected in the abscess culture, suggesting that broad-spectrum antibiotics may have been necessary. Thus, because progression from epididymitis to abscess formation is rare but possible, appropriate antibiotic selection for cases with severe inflammation remains a challenge for future research.

The observation period was longer in the poor-outcome group than in the favorable-outcome group, which may be attributed to two factors. First, in the poor-outcome group, a prolonged follow-up may have been conducted due to concerns about future testicular function. Second, long-term monitoring may have contributed to the identification of testicular atrophy, leading to poor outcomes. Testicular atrophy may develop over time rather than immediately after treatment. Some cases in this study were diagnosed as late as 6 months after onset. This prolonged observation suggests that poor outcomes may become evident only after an extended follow-up. Additionally, the variability in follow-up schedules highlights the need for standardized long-term monitoring to accurately assess the true incidence of poor outcomes.

This study identified fever, CRP levels, and decreased testicular blood flow as potential predictors of poor outcomes. Patients with these risk factors require long-term monitoring. Developing a predictive model for testicular function prognosis based on these factors remains a future challenge. A more accurate predictive model would enable early identification of high-risk cases and prompt initiation of treatment. Furthermore, optimizing antibiotic use based on these findings is necessary. Despite negative urinalysis and urine culture results, antibiotics are frequently administered, highlighting the need for antibiotic stewardship guidelines. Such guidelines would help curb the rise of antibiotic-resistant bacteria while ensuring effective treatment.

This study has some limitations, including the potential for selection bias in a single-center retrospective cohort study. Variability in patient backgrounds and treatment strategies, which may limit the generalizability of the findings, must be considered. Additionally, the sample size was relatively small, with 142 cases and only five cases with poor outcomes. The small number of poor-outcome cases may limit the conclusions that can be drawn regarding the risk factors and treatment strategies, necessitating caution when applying these results to broader patient populations. Furthermore, some data were missing, which may have influenced the analysis and interpretation of the results. Finally, our study lacked data on the long-term outcomes of testicular function, which is important for evaluating the future impact of epididymitis on fertility and endocrine function.

Prospective cohort studies and randomized controlled trials (RCTs) are needed to generate stronger evidence regarding the risk factors for poor outcomes and optimal treatment strategies for AE. A previous prospective study evaluated AE using ultrasonography at baseline and follow-up to assess disease progression in an adult cohort [24]. However, this study primarily focused on imaging findings and did not comprehensively analyze the risk factors for poor outcomes or treatment effects. Moreover, because the study was conducted in adults, its findings may not be directly applicable to pediatric AE. Therefore, pediatric-specific prospective studies are essential to refine risk stratification and clarify the role of clinical parameters such as fever, CRP levels, and decreased testicular blood flow.

Additionally, RCTs are essential to evaluate the necessity and optimal duration of antibiotic therapy as well as the effectiveness of different treatment approaches, including conservative management versus antibiotic use. Future large-scale, multi-center collaborative studies are required to gather data from a wider patient population and optimize treatment strategies. In addition, studies on long-term outcomes are essential to evaluate the sequelae of AE, particularly its impact on fertility and endocrine function. Collecting and analyzing longitudinal data on testicular function will be a crucial research agenda.

## Conclusion

In this study, we investigated the clinical characteristics and risk factors associated with poor outcomes in pediatric patients with AE. Our findings suggest that fever, CRP levels, and decreased testicular blood flow are predictors of poor outcomes. Additionally, the U-WBC and urine culture positivity rates were low, suggesting the need for more appropriate antibiotic use.

## Data Availability

No datasets were generated or analysed during the current study.

## References

[1] Rupp TJ, Leslie SW (2022) Epididymitis. StatPearls. StatPearls Publishing, Treasure Island, FL 28613565

[2] Chanchlani R, Acharya H (2023) Acute scrotum in children: a retrospective study of cases with review of literature. Cureus 15:e36259. 10.7759/cureus.36259. (**eCollection 2023 Mar**) 37073197 10.7759/cureus.36259 PMC10105644

[3] Tanaka K, Ogasawara Y, Nikai K et al (2020) Acute scrotum and testicular torsion in children: a retrospective study in a single institution. J Pediatr Urol 16(1):55–60. 10.1016/j.jpurol.2019.11.007 31874735 10.1016/j.jpurol.2019.11.007

[4] McConaghy JR, Panchal B (2016) Epididymitis. an overview. Am Fam Physician 94:723–726 27929243

[5] Somekh E, Gorenstein A, Serour F (2004) Acute epididymitis in boys: evidence of a post-infectious etiology. J Urol 171:391–394. 10.1097/01.ju.0000102160.55494.1f 14665940 10.1097/01.ju.0000102160.55494.1f

[6] Lau P, Anderson PA, Glacomatonio JM et al (1997) Acute epididymitis in boys: are antibiotics indicated? Br J Urol 79:797–800. 10.1046/j.1464-410x.1997.00129.x 9158522 10.1046/j.1464-410x.1997.00129.x

[7] Kido M, Nishida S, Nakamura K (2025) Pediatric epididymitis: a-20-year single-center experience of 61 cases. Pediatr Int 67:e15886. 10.1111/ped.15886 40033468 10.1111/ped.15886

[8] Evan MG, Alden B, Dominic F (2020) Salmonella epididymo-orchitis with testicular abscess in a newborn male. Int J Clin Case Rep Rev 2:5. 10.31579/2690-4861/015

[9] Chang CD, Lin JW, Lee CC et al (2016) Acute epididymo-orchitis-related global testicular infarction: clinical and ultrasound findings with an emphasis on the juxta-epididymal string-of-bead sign. Ultrasound Q 32(3):283–289. 10.1097/RUQ.0000000000000225 27556195 10.1097/RUQ.0000000000000225

[10] Hsu PC, Huang WJ, Huang SF (2017) Testicular infarction in a patient with spinal cord injury with epididymitis: a case report. J Rehabil Med 49:88–90. 10.2340/16501977-2174 27904914 10.2340/16501977-2174

[11] Akagawa Y, Kimata T, Akagawa S et al (2020) Optimal bacterial colony counts for the diagnosis of upper urinary tract infections in infants. Clin Exp Nephrol 24:253–258. 10.1007/s10157-019-01812-8. (**Epub 2019 Nov 12**) 31712943 10.1007/s10157-019-01812-8

[12] Raimund S, Dogan HS, Hoebeke P et al (2015) Urinary tract infections in children: EAU/ESPU guidelines. Eur Urol 67:546–558. 10.1016/j.eururo.2014.11.007. (**Epub 2014 Dec 2**) 25477258 10.1016/j.eururo.2014.11.007

[13] Sharma A, Minhas S, Dhillo WS et al (2021) Male infertility due to testicular disorders. J Clin Endocrinol Metab 106:e442–e459. 10.1210/clinem/dgaa781 33295608 10.1210/clinem/dgaa781 PMC7823320

[14] Haddad FH, Omari AA, Malkawi OM et al (2004) Patterns of testicular cytology in men with primary infertility: any change since the Gulf War? Acta Cytol 48:807–812. 10.1159/000326450 15581166 10.1159/000326450

[15] Ueda T, Yanagi M, Katsu A et al (2023) Obstructive azoospermia caused by epididymis injury with testicle rupture on the other side. Acute Med Surg 11:e919. 10.1002/ams2.919 38162166 10.1002/ams2.919 PMC10757826

[16] Vidal JD, Whitney KM (2014) Morphologic manifestations of testicular and epididymal toxicity. Spermatogenesis 4(2):e979099 26413388 10.4161/21565562.2014.979099 PMC4581048

[17] Joo JM, Yang SH, Kang TW et al (2013) Acute epididymitis in children: the role of the urine test. Korean J Urol 54:135–138. 10.4111/kju.2013.54.2.135. (**Epub 2013 Feb 18**) 23550228 10.4111/kju.2013.54.2.135 PMC3580304

[18] Sakellaris GS, Charissis GC (2008) Acute epididymitis in Greek children: a-3-year retrospective study. Eur J Pediatr 167:765–769. 10.1007/s00431-007-0584-y. (**Epub 2007 Sep 5**) 17786475 10.1007/s00431-007-0584-y

[19] Cristoforo TA (2021) Evaluating the necessity of antibiotics in the treatment of acute epididymitis in pediatric patients: a literature review of retrospective studies and data analysis. Pediatr Emerg Care 37:e1675–e1680. 10.1097/PEC.0000000000001018 28099292 10.1097/PEC.0000000000001018

[20] Redshaw JD, Tran TL, Wallis MC et al (2014) Epididymitis: a 21-year retrospective review of presentations to an outpatient urology clinic. J Urol 192(4):1203–1207. 10.1016/j.juro.2014.04.002 24735936 10.1016/j.juro.2014.04.002

[21] Gierup J, von Hedenberg C, Osterman A (1975) Acute non-specific epididymitis in boys. A survey based on 48 consecutive cases. Scand J Urol Nephrol 9:5–7. 10.3109/00365597509139905 1215845 10.3109/00365597509139905

[22] Gibbs EM, Baird BA, Frimberger D (2020) Salmonella epididymo-orchitis with testicular abscess in a newborn male. Int J Clin Case Rep Rev 2:5. 10.31579/2690-4861/015

[23] Lurz K, Santarelli S, Hager S et al (2021) Pediatric intratesticular abscess managed with a testicular sparing approach: a case report. Urol Case Rep 40:101873. 10.1016/j.eucr.2021.101873 34660205 10.1016/j.eucr.2021.101873 PMC8502707

[24] Pilatz A, Wagenlehner F, Bschleipfer T et al (2013) Acute epididymitis in ultrasound: results of a prospective study with baseline and follow-up investigations in 134 patients. Eur J Radiol 82:e762–e768. 10.1016/j.ejrad.2013.08.050 24094645 10.1016/j.ejrad.2013.08.050

[25] Alharbi B, Rajih E, Adeoye A et al (2019) Testicular ischemia secondary to epididymo-orchitis. A case report. Urol Case Rep 27:100893. 10.1016/j.eucr.2019.100893 31687342 10.1016/j.eucr.2019.100893 PMC6819811

[26] Yusuf G, Sellars ME, Kooiman GG et al (2013) Global testicular infarction in the presence of epididymitis. J Ultrasound Med 32:175–180. 10.7863/jum.2013.32.1.175 23269723 10.7863/jum.2013.32.1.175

[27] Zhong W, Virk A, Leslie S et al (2021) A challenging case of surgical management of a single ischemic testicle from severe epididymo-orchitis. Urol Case Rep 37:101610. 10.1016/j.eucr.2021.101610 33680852 10.1016/j.eucr.2021.101610 PMC7918267

[28] Wang Z, Qiu M, Gao X et al (2023) Testicular ischemia secondary to acute epididymitis: a case report. Medicine (Baltimore) 102:e33843. 10.1097/MD.0000000000033843 37335700 10.1097/MD.0000000000033843 PMC10194603

[29] Sukhotnik I, Meyer G, Nativ O et al (2008) Effect of allopurinol on germ cell apoptosis following testicular ischemia-reperfusion injury in a rat. Pediatr Surg Int 24:61–66. 10.1007/s00383-007-2042-3 17985141 10.1007/s00383-007-2042-3

[30] Taati M, Moghadasi M, Dezfoulian O et al (2012) The effect of ghrelin pretreatment on epididymal sperm quality and tissue antioxidant enzyme activities after testicular ischemia/reperfusion in rats. J Physiol Biochem 68:91–97. 10.1007/s13105-011-0122-2 21994044 10.1007/s13105-011-0122-2

